# Self-monitoring and psychoeducation in bipolar patients with a smart-phone application (SIMPLe) project: design, development and studies protocols

**DOI:** 10.1186/s12888-015-0437-6

**Published:** 2015-03-20

**Authors:** Diego Hidalgo-Mazzei, Ainoa Mateu, María Reinares, Juan Undurraga, Caterina del Mar Bonnín, José Sánchez-Moreno, Eduard Vieta, Francesc Colom

**Affiliations:** Bipolar Disorders Unit, Neurosciences Institute, Hospital Clínic de Barcelona, IDIBAPS, CIBERSAM, Universitat de Barcelona, Villarroel 170, 08036 Barcelona, Catalonia Spain; Department of Psychiatry and Psychology, Institute of Neuroscience, Hospital Clinic, University of Barcelona, IDIBAPS, CIBERSAM, Barcelona, Catalonia Spain; Department of Psychiatry, Facultad de Medicina Clinica Alemana Universidad del Desarrollo, Santiago, Chile

**Keywords:** Bipolar disorder, Psychoeducation, Monitoring, Smartphones, Self-management

## Abstract

**Background:**

New technologies have recently been used for monitoring signs and symptoms of mental health illnesses and particularly have been tested to improve the outcomes in bipolar disorders. Web-based psychoeducational programs for bipolar disorders have also been implemented, yet to our knowledge, none of them have integrated both approaches in one single intervention. The aim of this project is to develop and validate a smartphone application to monitor symptoms and signs and empower the self-management of bipolar disorder, offering customized embedded psychoeducation contents, in order to identify early symptoms and prevent relapses and hospitalizations.

**Methods/design:**

The project will be carried out in three complementary phases, which will include a feasibility study (first phase), a qualitative study (second phase) and a randomized controlled trial (third phase) comparing the smartphone application (SIMPLe) on top of treatment as usual with treatment as usual alone. During the first phase, feasibility and satisfaction will be assessed with the application usage log data and with an electronic survey. Focus groups will be conducted and technical improvements will be incorporated at the second phase. Finally, at the third phase, survival analysis with multivariate data analysis will be performed and relationships between socio-demographic, clinical variables and assessments scores with relapses in each group will be explored.

**Discussion:**

This project could result in a highly available, user-friendly and not costly monitoring and psychoeducational intervention that could improve the outcome of people suffering from bipolar disorders in a practical and secure way.

**Trial registration:**

Clinical Trials.gov: NCT02258711 (October 2014).

## Background

Bipolar disorder is a frequent condition in the general population with a high morbimortality [1]. It is characterized by dysfunctional episodic fluctuations between different mood phases ranging from depression to manic episodes with patients presenting frequent interepisodic subsyndromal symptoms. Frequently, people with this condition have a lack of insight about their diagnosis and symptoms, especially regarding manic phases, which leads to poor prognosis [2,3].

Besides the pharmacological treatment, adjunctive psychological interventions have shown to improve the long-term outcome of the disorder [4], although, taking into account the limited resources currently available, their extended implementation is still difficult and costly [5].

Among psychotherapeutic interventions, psychoeducational programs have proven to be cost-effective in helping patients recognize early signs or symptoms and adopt behavioral measures to prevent full-blown episodes which are frequently associated with a high morbidity and more hospital admissions [6,7]. Accordingly, there is an increased need to make this intervention more widely available, without compromising its quality [4].

On the other hand, the wide use and availability of new technologies such as the Internet have been successfully adopted in mental health contexts. Using these technologies in patient’s assessments and interventions have proved their efficacy and reliability as well as their good acceptability from the patient’s perspective [8,9]. The potential improvement in accessibility to healthcare in patients with disabilities or patients living in rural or other remote areas (i.e. telemental health), as well as the lower costs when compared to conventional interventions, makes them an attractive complement to standard treatment [10-13].

Furthermore, the progressively reduced costs and consequent widespread accessibility to mobile phones with internet connection (smartphones) opens an unlimited number of opportunities to the mental health field. In industrialized countries, these devices have become a very popular way of interacting with each other and with the environment. As an example, in Spain, a recent study of the National Institute of Statistics revealed that almost 70% of the population have internet access and in the majority, through a mobile device [14]. This is a growing phenomenon and includes developing countries as well. According to eMarketer Inc., a company which studies technology market trends, by 2017 one-third of all the population around the globe will be using a smartphone [15].

In addition, the constant improvement of portability and benefits of mobile devices allows the quick and continuous collection of relevant users’ information at a low-cost. Data on subject’s activity and interests, through embedded sensors and mobile usage patterns respectively, are collected and complemented with information from other digital services such as social networks, e-mails and internet search patterns. All this information that has been denominated “big data” are integrated, analyzed by data mining and results are used to determine individual user behavior and interests patterns by predictive analytics, which now are commonly used for commercial purposes [16]. Accordingly, there is an increasing interest in medicine and especially in mental health to explore the possibilities and potential applications of this underutilized data [17-20].

The potential of the great amount of data collected by the patient’s smartphones, its analysis and potential applications in treatment interventions are leading the way to the (so-called) “Personalized Medicine” era. Given the diverse types of presentations, course and response to treatments in mental disorders, including new technologies has long been promoted as the obligatory next step in different medical disciplines, and especially in psychiatry [21-24]. However, one major concern about this approach is the potential threats to patient’s privacy and consequent utilization of this data with others intends, if it is transmitted, processed or stored insecurely [25,26].

Several projects have tested the benefits of these new technologies for the treatment of bipolar and psychotic disorders using either online monitoring of signs and symptoms [27,28] or web-based psychoeducational programs [29-32], yet to our knowledge, none of them have integrated both approaches in one single intervention.

The current technology available makes technically easier to integrate into patient’s life a comfortable, simple, time-unconstrained, user-friendly, economical and non-invasive method of registering and monitoring relevant signs and symptoms and provide continuous self-management psychoeducational contents tailored to the specific needs of each individual on the basis of these data registered on their own smartphones [33]. Additionally, this approach could contribute to better understand the pathoetiology as well as prodromal behavior patterns prior to a relapse in bipolar disorders, integrating objective and subjective data with other clinical correlates [17].

We hypothesized that, combining a signs and symptoms monitoring system with continuous feeds of tailored psychoeducational in a single smartphone application as an adjunctive intervention to usual treatment, would add efficacy in preventing relapses, suicide attempts and health resources consumption in bipolar patients improving their overall prognosis.

The aim of this study is to develop and clinically validate a smartphone application to monitor symptoms and signs in bipolar patients, offering customized embedded psychoeducation contents and empower the self-management of their disorder in order to prevent relapses and hospitalizations. Secondary objectives are to explore other clinical benefits among the smartphone application users such as improvements in biological rhythms, manic/hypomanic and depressive symptoms, quality of life, suicide attempts and pharmacological treatment adherence.

## Methods and design

**Figure 1:**
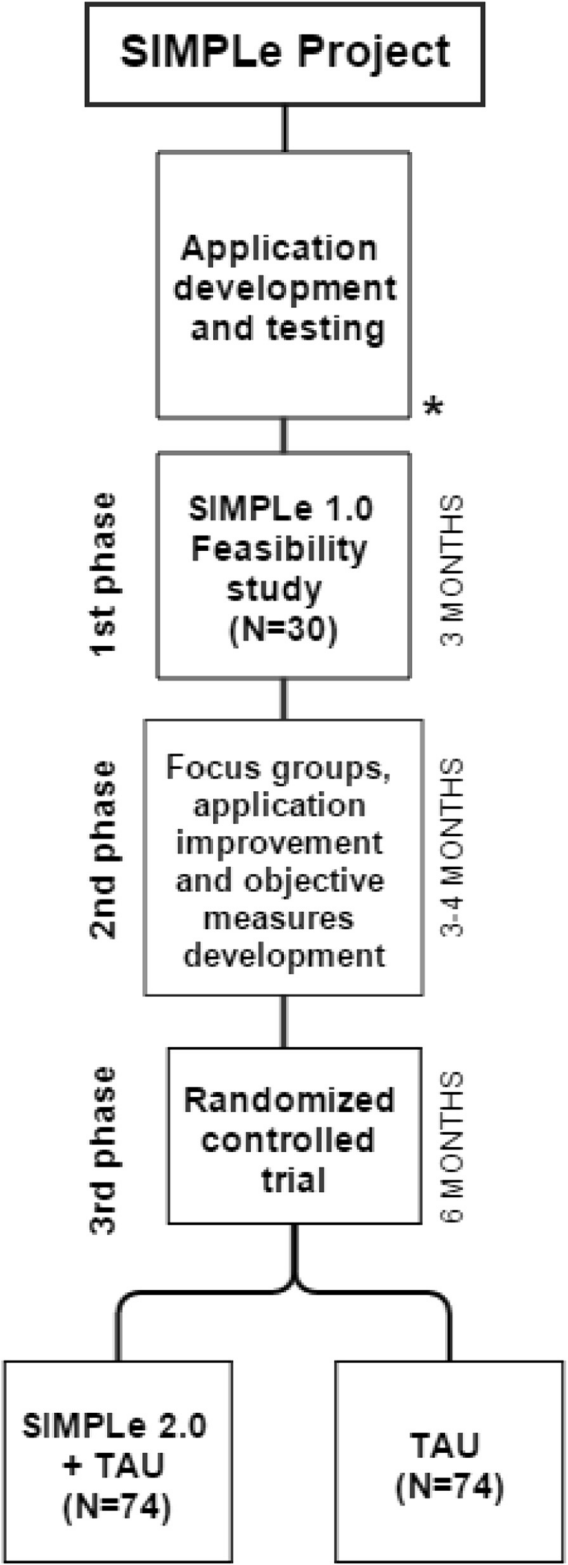
**The figure shows a flowchart of the SIMPLe project phases (left) and their expected duration (right).** *Current status of the study. TAU = Treatment as usual.

Given the incipient nature of the field, we adopted an eclectic approach considering and combining multiple guidelines and recommendations about developing internet and mobile interventions for mental health [34-36]. In order to consider technical aspects and an adequate clinical validation while including patients preferences and safety along the process, it was determined that the project will be carried out in three complementary phases, which will include two clinical studies. The studies will follow the Consolidated Standards of Reporting Trials (CONSORT) guidelines to describe, report and publish the results [37] (Figure 1).

### Application development

During one year (May 2013 to May 2014) a collaborative team of specialists in bipolar disorder (psychiatrists and psychologists from the Bipolar Disorders Program of the Hospital Clínic of Barcelona) and software engineers as well as graphical designers held periodical meetings in order to determine the design of an online tool to monitor and register signs and symptoms of bipolar disorder and offer personalized psychoeducation contents. At the end of this process, the team resolved to develop a smartphone application with the following characteristics: user-friendly, non-stigmatizing and sensitive enough to detect mood changes in order to give customized psychoeducational feedback based on an existing psychoeducation group treatment manual [38], which has been previously tested by the Barcelona Bipolar Disorders Program [6].

Several evidence-based methods for self-mood assessment were considered taking into account the aims of the project. We chose 5 daily key questions balancing sensitivity and privacy, which are intended to be answered in a few seconds with the aid of an animated graphical interface (i.e. mood, energy, sleep time, irritability and medication adherence). If a relevant mood change is detected by means of an algorithm that takes into consideration the previous week mean score of the daily test, the user is requested to answer a more comprehensive second test based on the DSM-5 [39] criteria for mania, hypomania or depression, along with some extra questions regarding substance use and suicide risk. If the patient gives an affirmative answer to the question that explores suicidal ideation, an automatic e-mail message is sent to the mental healthcare team and in addition, an immediate call to emergency services is offered. Otherwise, if the subject is stable during their daily assessments, this DSM5 based test is requested to be answered only once a week.

**Figure 2:**
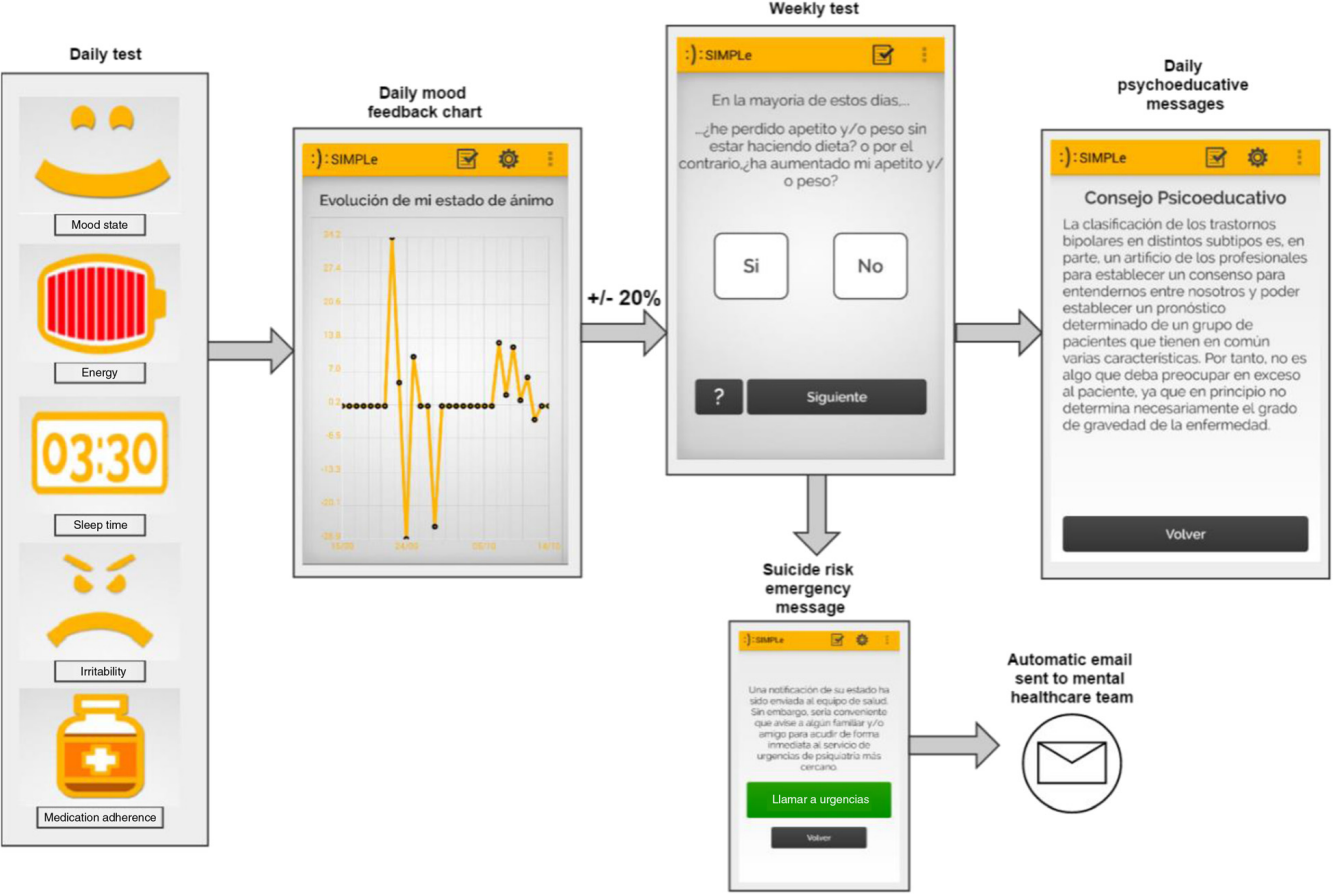
**The chart shows the way in which the application SIMPLe 1.0 gives psychoeducative messages based on the answers of daily and weekly tests.**

Furthermore, the application will give feedback with a graphic showing daily mood changes and the user will be able to review the results of previous tests. Personalized daily psychoeducative messages will be sent based on the patient’s answers. The user will be able to customize the best time of the day to answer the test and to receive the psychoeducative messages at his or her convenience (Figure 2).

Simultaneously, a web-based interface will be developed to allow the mental healthcare team to follow and monitor patient status.

**Figure 3:**
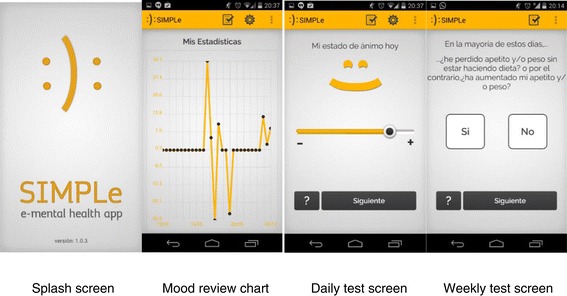
**Screenshots of SIMPLe 1.0 running in an Android Smartphone.**

The collaborative team decided that it was necessary a first study to determine the acceptability and validity of an application based solely on subjective information (SIMPLE 1.0), prior to the addition of objective monitoring data modules (SIMPLE 2.0) for three reasons: 1. In order to give patients active participation in the development of the application through focus groups and satisfaction surveys based on the experience with the first SIMPLE version and adapt the application to their preferences; 2. To test data safety and confidentiality issues with a smaller sample; 3. To adjust possible mood sensitivity issues with the daily and weekly questionnaires which were not previously validated in a real-world clinical setting (Figure 3).

### Study design and phases

The studies to evaluate the smartphone application will be carried out in three different but complementary phases as detailed below.

- **First phase:** A three months lasting feasibility study will be conducted in order to evaluate the use, reliability and patient satisfaction with symptom monitoring (subjective information). Patients under usual treatment will use a smartphone with the SIMPLe version 1.0 application installed. The intervention will be consecutively offered to 30 stable adults (>18 years) fulfilling the inclusion criteria described below. An informed consent will be handled and explained and must be signed in order to participate. Sociodemographic data and standardized clinical as well as functional assessments will be administered at baseline and monthly for three months. Assessments will include present manic symptoms using the Young Mania Rating Scale (YMRS) [40,41], depressive symptoms using the Hamilton depression Rating Scale (HDRS) [42,43], biological rhythms using the biological rhythms interview of assessment in neuropsychiatry (BRIAN) [44,45] and treatment adherence using the Morisky Green 8-item test [46]. A smartphone device with the SIMPLe 1.0 application pre-installed, will be given to the participants for free during the study with the mandatory condition that it has to be their main mobile-phone during the next 3 months. In the case the patient accepts to participate but doesn’t want to switch from his/her current smartphone, he/she will be offered the possibility to download the SIMPLe 1.0 application from the Application Store and a username and password will be given. The subject will receive a customizable automatic reminder in their smartphone to answer both daily and weekly questionnaires based on DSM-5 criteria for a manic, hypomanic, mixed or depressive episodes. The data obtained will be registered online. During this period of time, the subjects will receive daily customized psychoeducative messages based on the information collected by the application using an algorithm designed by our team. Feasibility and satisfaction will be assessed with the application usage log data and with an electronic survey. Reliability will be analyzed comparing the clinical assessments made by a psychiatrist and the information about mood state collected by the application
- **Second phase:** Three different focus groups (patients, psychiatrists and psychologists, software engineers and graphical designers) of 3 sessions each will be held to discuss the experience with the SIMPLE 1.0 app and recollect suggestions and bugs. Additionally, individual personal interviews with each SIMPLE 1.0 user will also collect qualitative information about the application and suggestions. Taking into consideration the information collected from participants in the first phase, the application will be adapted and improved with the addition of objective information (signs) monitoring modules using mobile usage parameters and the built-in actimetry sensors.
- **Third phase:** A six months randomized controlled trial with an intervention and a control arm including 74 patients each. An initial evaluation in both groups will be carried out recollecting sociodemographic data and using standardized clinical as well as functional assessments. This evaluation will be repeated at three, six and twelve months from baseline. The assessments will include present manic symptoms using the Young Mania Rating Scale (YMRS), depressive symptoms using the Hamilton depression Rating Scale (HDRS), functional impairment using the Functioning assessment short test (FAST) [47], biological rhythms using the biological rhythms interview of assessment in neuropsychiatry (BRIAN), quality of life measured by World Health Organization quality of life assessment (WHOQOL-BREF) [48] and treatment adherence using the Morisky Green 8-item test. During the follow-up the number of relapses, inpatient admissions, outpatient consultations, emergency rooms consultations and suicide attempts will be registered.

1. **Intervention arm (SIMPLe 2.0 + TAU):** The experimental group will use the application SIMPLe 2.0 and will keep receiving their usual pharmacological treatment. A smartphone device with the SIMPLe 2.0 application pre-installed, will be given to the participants for free during the study with the mandatory condition that it has to be their main mobile-phone during the next 3 months. In the case the patient accepts to participate but does not want to switch from their current smartphone, they will be offered the possibility to download the SIMPLe 2.0 application from the Application Store and a username and password will be assigned. The self-mood assessments will adopt a similar approach to the SIMPLe 1.0 app. Additionally this information will be complemented with automatically recorded patients activity data which will include smartphone usage, telephone calls made (number of independent calls and total time), social network apps usage, and physical activity measured by the built-in actimeter. Patients will receive daily psychoeducative messages based on the data recollected by the application. Relapse risk will be calculated using an algorithm including both subjective and objective data. The clinical team will be contacted with an e-mail if there is moderate or high risk for an oncoming episode and the patient will be offered the outpatient or emergency for assistance.
2. **Treatment as usual arm (TAU):** The treatment as usual (TAU) group will be followed-up with their standard treatment following the Barcelona Bipolar Disorders Program protocol [49].

### Participants

#### Sample recruitment

The participation in the study will be offered to stable bipolar patients (YMRS ≤ 8, HDRS ≤ 6 for at least one month prior to study entry) who had experienced at least one hypomanic, manic or depressive relapse during the 12 months prior to the study entry. All the participants will be adult patients attending the outpatient mental health clinic of the Hospital Clinic of Barcelona. All participants in both groups must read and agree with the terms and sign an informed consent prior to their inclusion in the study.

The following selection criteria will be considered for the first and third phase:

#### Inclusion criteria

1. Age from 18 to 65 years old, 2. Patients with a current diagnosis of Bipolar disorder type I or type II according to DSM-5 criteria and confirmed with a semi-structured interview (SCID), 3. Hamilton Depression Scale score under or equal to 8 during the last month, 4. Young Mania Rating Scale score under or equal to 6 during the last month, 5. No history of relapses during the previous 3 months but at least 1 relapse during the last year, and 6. Availability of an unlimited smartphone data plan during the 12 following months to avoid extra costs for the participants when using the application.

#### Exclusion criteria

1. Lack of skills to use the offered smartphone or unwillingness to learn them, 2. FAST score above or equal to 20, 3. Current or recent (within the last four years) participation in psychoeducation groups or in individual psychological treatment for the management of bipolar disorder that includes psychoeducational interventions, 4. Obsessive-compulsive disorder according to DSM-5 criteria, 5. Concomitant severe medical condition, 6. Current pharmacological treatment with long-acting antipsychotics and 7. Pregnancy.

### Randomization, data anonymization and blinding

A 5 digits random identification number (IDN) will be generated for all the participants throughout all the phases of the study. The cross-reference of this identification number and the patient identity will be encrypted and stored in a database file. Those patients using the smartphone application will be identified only by the IDN, which will be also the username to access the application. The patient will be asked by the application to enter the username and a password once per smartphone session if he/she is using a current security method to block the phone (code, pattern, face or finger recognition), otherwise the user will have to enter the username and password every time the SIMPLE app is opened.

During the third phase, an independent researcher using the site randomizer.org [50] will randomize the sample to the intervention group or to the control group. Interviews, assessments and focus groups related to the study will be carried out by independent clinicians. Due to ethical considerations, this blinding process will be interrupted if a risk situation is detected through the application during any phase of the study. In this case, the subject will be identified and their therapists will be informed immediately. Exclusion from the study will be discussed depending on the circumstances.

### Power and sample calculation

We adopted a conservative approach for the first phase of the study to ensure more close qualitative feedback. Accordingly we calculated a sample size of 30 based in the recommendations for internal pilot studies from Kieser&Friede [51]. For the third phase, a significance level of 0.05, power of 0.8 and a minimal detectable difference of 0.5 and an expected drop-out of 15% was set, which resulted according to the design in two arms of 74 patients each [52].

### Statistical analysis

During the first phase, a descriptive analysis of socio-demographic and clinical variables will be conducted, including mean age, sex, marital status, employment status, family psychiatric history, follow up time, number of episodes, predominant polarity, substance abuse and comorbid medical and psychiatric diagnoses. Feasibility will be evaluated analyzing the frequency of daily and weekly tests completed, if important differences in baseline characteristics between adherent and not adherent users are detected, this will be further controlled by regression analyses. The results of the satisfaction survey will be estimated analyzing the percentage of answers and the differences among the different categories using Chi-square analysis. Additionally, a Pearson correlation analysis will be carried out between the results of the assessments during the 3 month period and the results of the application tests to evaluate the clinical reliability between the two methods.

At the third phase, survival analysis with multivariate data analysis will be carried out to assess several covariates simultaneously and explore relationships between socio-demographic, clinical variables and assessments scores with relapses in each group (Kaplan-Meier). Similarly, differences among the different assessments will be analyzed with multivariate survival analysis (Cox regression). Similarly as in the first phase, a Pearson correlation analysis will be carried out between the results of the assessments and completed application tests. All the analyses will be two-tailed with an alpha set at p < 0.05. Additionally, last observation carried-forward intention to treat analyses will be performed.

### Ethical and safety considerations

One of the main concerns during the development of the application was patient safety. Hence, the application incorporated a function which automatically notify the mental health team about suicide risk and give the patients the possibility to make a call to the emergency services from their own smartphone. Depending on the severity of the symptoms or if suicidal risk is detected, the blinding of the protocol will be broken and the case will be immediately discussed with their therapists.

In order to ensure participants’ confidentiality during the study and the transmission of personal data we adopted a security protocol using the IDN, as it has been previously described, which is in accordance to the local spanish laws (Ley orgánica de Protección de Datos de Carácter Personal (15/1999)). The username and password for the application will be requested every time the application is used if the user didn’t active a smartphone lock system, otherwise it could be used without entering the username and password. Additionally, the study protocol was evaluated and approved (Reg. HCB/2014/0403) by the Hospital Clinic of Barcelona ethical committee to ensure is accordance to the Helsinki Declaration. The study was registered at Clinicaltrials.gov with the identifier NCT02258711 on October 2014.

### Trial status

At the time of the elaboration of this manuscript the development phase of SIMPLe 1.0 was concluded and a testing process of the application was being carried out intensively by 4 software engineers, 7 psychologists and 7 psychiatrists before the initiation of the first phase of the study with patients.

## Discussion

Even when the current pharmacological and psychological treatments available for bipolar disorder can certainly improve the outcome and quality of life of bipolar patients, the access to such specialized treatments are concentrated in few reference centers around the globe, which can hardly cover the needs of a disease with a prevalence of almost 2% of the world population [53].

On the other side, during recent years new technologies have facilitated our life in many ways (e.g. internet shopping, traveling and communication), although its implementation in mental healthcare has been slow and underdeveloped when compared to other areas (i.e. commercial). Hence, using this technology in mental healthcare could improve the lives of thousands of people suffering from mental disorders at a very low cost and in a convenient, comfortable and secure way for patients.

Among other treatments, our research group has been promoting and providing high-quality evidence-based psychoeducational programs for more than 15 years [54,55]. However, our current face-to-face approach to psychoeducation excludes many patients who could benefit from it, such as those living in remote locations, those with incompatible schedules, patients who don’t want to attend a group therapy, etc. One of the advantages of new technologies, and specifically smartphones, is that it is possible to overcome these difficulties in a cost-efficient way through them. The tested application will represent a user-friendly, low cost and a potentially efficacious way to provide self-monitoring and personalized training to bipolar patients as an add-on to usual pharmacological and psychological care. Another benefit of this device is to include measures to facilitate safety procedures if any risk (i.e. suicidal thoughts) is detected. Furthermore, it can have a positive impact on associated healthcare costs.

There are some potential risks and limitations of this project: The fact that the patient will interact with a device instead of a therapist could decrease the level of compromise with the program. Furthermore, despite the fact that most people can have access to smartphones there is still a requirement of a minimum range of skills to use this kind of devices and operating systems. In this regard, many patients with bipolar disorder have some degree of cognitive or functional impairment [56], which could limit their capacity to use the device. In fact, a recent study has proven that patients with bipolar disorder show a poorer knowledge of computer use devices, internet and social media when compared with matched healthy controls [57].

Regarding the limitations of the study itself, the intervention is compared to treatment as usual and patients are not blinded to the intervention that they are receiving. It is almost impossible to create a “placebo smartphone application”. Another fact is that all participants will be recruited from a specialized mental healthcare center in bipolar disorder, which could limit the generalizability of the results to primary care contexts due to the clinical characteristics of the sample and the intensive healthcare character of the program.

If the first steps of the project show encouraging results, many of the mentioned limitations could be circumvented in the future including active comparators such as conventional psychoeducational programs and involving non-specialized settings in future efficiency studies.
